# Delta-Aminolevulinate dehydratase and glutathione peroxidase activity in Alzheimer's disease: a case-control study

**DOI:** 10.17179/excli2019-1749

**Published:** 2019-09-25

**Authors:** Quelen Iane Garlet, Maria Vaitsa Losh Haskel, Romaiana Picada Pereira, Weber Cláudio Francisco Nunes da Silva, João Batista Teixeira da Rocha, Cláudia Sirlene Oliveira, Juliana Sartori Bonini

**Affiliations:** 1 Departamento de Farmacologia, Instituto de Ciências Biológicas, Universidade Federal de Rio Grande, Rio Grande/RS, Brazil; 2 Departamento de Fisiologia Humana, Universidade Federal do Rio Grande do Sul, Porto Alegre/RS90040-060, Brazil; 3 Departamento de Química, Universidade Estadual de Ponta Grossa, Ponta Grossa/PR, Brazil; 4 Universidade Estadual do Centro-Oeste, Campus CEDETEG, Departamento de Farmácia, Guarapuava/PR, Brazil; 5 Departamento de Bioquímica e Biologia Molecular, Centro de Ciências Naturais e Exatas, Universidade Federal de Santa Maria, Santa Maria/RS, Brazil; 6 Programa Pós-Graduação Stricto Sensu em Biotecnologia Aplicada a Saúde da Criança e do Adolescente, Instituto de Pesquisa Pelé Pequeno Príncipe, Curitiba/PR, Brazil; 7 Faculdades Pequeno Príncipe, Curitiba/PR, Brazil

**Keywords:** Alzheimer's disease, delta-ALA-D, Gpx, AD marker, CDR, MMSE

## Abstract

Alzheimer's disease (AD) is a neurodegenerative pathology that affects elderly people all over the world. Several studies have demonstrated that oxidative stress is an aggravating factor for AD development and progression. Therefore, this study aimed to evaluate the activity of two oxidative stress markers, glutathione peroxidase (GPx) and δ-aminolevulinate dehydratase (δ-ALA-D), as well as correlate them with blood metal levels and AD progression. For this purpose, 88 elderly individuals were divided in two groups: AD group (34 patients diagnosed with AD) and control group (34 subjects paired by age with the AD group). The Mini-Mental State Examination and the Clinical Dementia Rating (CDR) were used as tools to classify the AD progression. GPx and δ-ALA-D activities were measured in all subjects through blood tests. Both enzymes' activities were decreased in AD patients when compared to the age-matched control group, regardless of the CDR. Moreover, GPx activity was positively correlated with selenium levels in the blood; and the δ-ALA-D activity was negatively correlated with blood copper levels. Taken together, our results indicated that, for the first time, blood δ-ALA-D activity was significantly inhibited in AD patients. While literature reports conflicting data regarding GPx activity in AD patients, the δ-ALA-D activity seems to be a more consistent tool to be applied as an earlier AD marker.

## Introduction

Alzheimer's disease (AD) is a neurodegenerative illness usually characterized by progressive memory loss and the degeneration of, at least, another cognitive function such as language, attention or reasoning (Dubois et al., 2007[18]; Selkoe, 2001[49]). However, pieces of evidence have indicated that patients with mild or without cognitive impairment can also display other AD-related pathological alterations (Bennett et al., 2006[5]; James and Bennett, 2019[24]; Schneider et al., 2009[48]). The AD development is multifactorial depending on genetic and/or environmental factors; however, its pathogenesis remains largely unknown and the treatments do not modify its progression (Ballard et al., 2011[4]; Malhotra, 2018[28]; Trinh et al., 2003[51]).

Studies about the mechanisms involved in the AD pathogenesis point out that inflammation and oxidative stress play an important role in AD development (Castora, 2019[10]; Halliwell, 2006[22]; Lin and Beal, 2006[27]). The brain tissue is particularly susceptible to oxidative stress damage due to its high metabolic demand, consuming about 20 % of the inspired oxygen. It has a high concentration of polyunsaturated fatty acids, weak antioxidant machinery and high levels of iron and excitatory neurotransmitters (Di Domenico et al., 2017[17]; Halliwell, 1992[23]; Markesbery and Carney, 1999[29]). Regarding physiological conditions, the organism exploits a sophisticated enzymatic and non-enzymatic antioxidant defense system, protecting itself from oxidative damage progression (Halliwell, 1996[21]). 

The micronutrients iron (Fe), copper (Cu) and selenium (Se) are needed for the antioxidant enzymes optimal functioning, e.g., glutathione peroxidase (GPx) or superoxide dismutase (SOD), (Angeli and Conrad, 2018[1]; Flohe et al., 1973[20]; Rocha et al., 2017[42]; Younus, 2018[56]). These metals have a pivotal role in synapse regulation and are essential cofactors participating in various enzymatic activities. Consequently, an alteration in the concentration of these elements may induce the exacerbation of several diseases, including neuronal pathologies (Prohaska, 1987[39]; Scheiber et al., 2014[47]; Solovyev, 2015[50]; Ward et al., 2014[55]). Indeed, a previous study reported that AD patients showed a CDR-dependent increased level of Fe and Cu when compared to control subjects (Vaz et al., 2018[53]).

The δ-aminolevulinate dehydratase (δ-ALA-D) or porphobilinogen synthase (PBG-synthase) is a thiol-containing enzyme that can be oxidized under oxidative stress conditions (Rocha et al., 2012[43]). Accordingly, δ-ALA-D inhibition results in high concentration of its substrate (5-aminolevulinic acid), which has a pro-oxidative effect (Costa et al., 1997[12]; Demasi et al., 1996[16], 1997[15]; Rocha et al., 2012[43]). In this context, da Silva et al. (2007[13]) suggested that δ-ALA-D inhibition can be used as an oxidative stress index. However, literature lacks conclusive and accurate data about the correlation among metals present in the body, enzyme activity and AD. Therefore, we aim to elucidate whether the enzymes GPx and δ-ALA-D and the micronutrients Cu, Fe, and Se play a role in the AD progression, providing new information that could indicate alternative or auxiliary approaches to treat and/or detect this neurodegenerative disease.

## Materials and Methods

### Population and cognitive validation

This is a case-control, cross-sectional study. The study was authorized by the Human Research Ethics Committee of the Universidade Estadual do Centro-Oeste (61111316/201). Data were collected from June 2013 to December 2014. The samples consisted of 88 elderly individuals divided in two groups: (1) *AD group*: This group had 34 individuals diagnosed with AD who were registered in the 5^th^ Department of Health of Guarapuava city, Paraná State, Brazil. The elderlies invited to participate in this study had the AD diagnosis confirmed according to the criteria of the National Institute of Neurological and Communicative Disorders and Stroke and Alzheimer Disease and Related Disorders Association. The Mini-Mental State Examination (MMSE) and the Clinical Dementia Rating (CDR) were combined to better track and classify cognitive disorders. These methodologies allowed the AD progression classification in mild dementia (CDR1 and MMEM score ranging from 20 to 27 for subjects that attended > 4 years of school or from 16 to 21 for subjects that attended ≤ 4 years of school), moderate dementia (CDR2 and MMEM score ranging from 12 to 19 for subjects that attended > 4 years of school or from 8 to 15 for subjects that attended ≤ 4 years of school) and severe dementia (CDR3 and MMEM score < 11 for subjects that attended > 4 years of school or < 7 for subjects that attended ≤ 4 years of school). (2)* Control group*: This group had 34 individuals (MMEM score >27 for subjects that attended > 4 years of school or >21 for subjects that attended ≤ 4 years of school) carefully paired to the AD group. According to Vaz et al. (2018[53]), the pairing criterion was organized considering the year of birth, sex, if the subject was a smoker or not, diabetes mellitus diagnosis and high blood pressure.

### Enzymatic assays

To access the GPx and δ-ALA-D activity, we performed assays using blood (5 ml). The blood samples were collected at the participants' homes and they respected an 8-hour fasting time. Immediately after, the blood was transferred to a tube containing heparin. Blood samples were diluted (1:3) in distilled water followed by agitation on ice to provide total hemolysis. 

### δ-ALA-D activity

The δ-ALA-D activity was measured according to the method described by Berlin and Schaller (1974). Enzyme activity was determined by the rate of product (porphobilinogen-PBG) formation. The reaction was started by adding the substrate (5-aminolevulinic acid) followed by incubation at 37 ºC for 90 min in the presence or absence of the reducing agent dithiothreitol (2 mM DTT). The reaction was stopped by the addition of trichloroacetic acid (TCA) 10 % containing HgCl_2_ 0.05 M. PBG was measured with Ehrlich's reagent using the molar absorption coefficient of 6.1 x 10^4 ^for Ehrlich-PBG salt. Specific enzymatic activity was expressed as nmol PBG/h/ml (blood hemolysate).

### GPx activity

GPx activity was assessed according to the method described by Paglia and Valentine (1967[35]). The conversion of NADPH to NADP^+^ was monitored at 340 nm for 2 min. Enzyme activity was expressed as µmol of NADPH oxidized per min/ml (blood hemolysate) using an extinction coefficient of 6.2×10^6^ for NADPH.

### Statistical analysis

The results are presented as mean ± standard error mean (SEM) or median ± interquartile interval (non-parametric data). Homogeneity of variance and normality were evaluated by Levene and Shapiro-Wilk tests, respectively. Parametric data were submitted to unpaired two-tailed t-test and non-parametric data were analyzed by Mann-Whitney test. Correlations were analyzed by the Pearson correlation method. Statistical analysis was carried out using SigmaPlot 11.0 software or GraphPad Prism version 6.01 and the minimum significance level was set at p < 0.05.

## Results

### Cognitive validation

**Figure 1:**
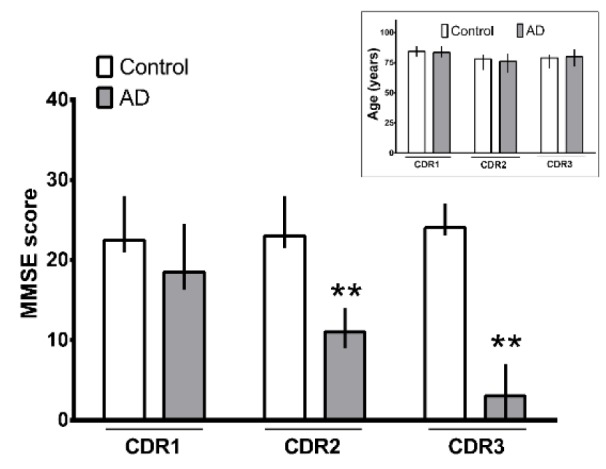
MMSE score and age (insert graph) from control subjects and Alzheimer Disease (AD) patients subdivided by CDRs. **p<0.001 from respective control group

We evaluated the subjects from the control group and AD patients using MMSE scores, CDR and age data to classify them regarding its AD stage (Figure 1(Fig. 1)). Raw data from subjects can be assessed in supplementary material (Supplementary Table 1). Age parameter was homogeneous among groups (p> 0.05) and patients in the CDR1 stage had similar MMSE scores to the respective control group. On the other hand, AD patients in CDR2 and CDR3 stages showed a significant decrease in the MMSE score compared to the respective control group (p<0.001, Mann-Whitney unpaired two-tailed test).

### Enzymatic activity

**Figure 2:**
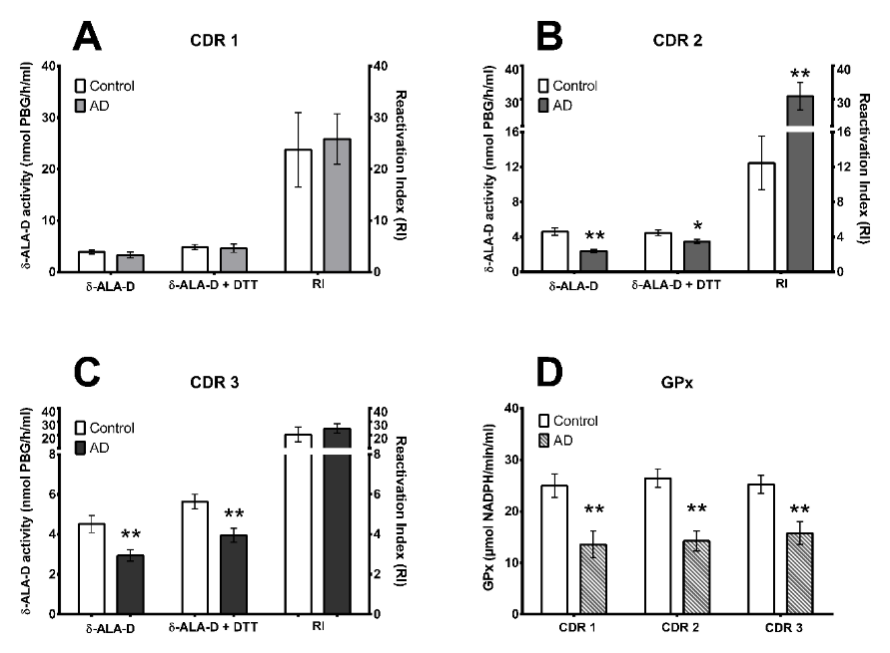
Blood δ-Amino δ-ALA-D and GPx activity in different stages of Alzheimer Disease (AD). δ-ALA-D activity with or without DTT addition and its reactivation index (RI) in AD stages CDR1, CDR2, and CDR3 (A, B and C, respectively) and GPx activity in AD stages CDR1, CDR2, and CDR3 (D). *p< 0.05 or **p< 0.001 from control group

We assessed the enzymes δ-ALA-D activity, which plays a role in the heme biosynthesis pathway and GPx which is involved in oxidative damage repair (Figure 2(Fig. 2)). Raw data from subjects can be assessed in supplementary material (Supplementary Table 1). We detected a decrease in δ-ALA-D activity in patients ranging from moderated (CDR2) to advanced (CDR3) AD stage (Figure 2A, B, and C(Fig. 2), p< 0.05 or p< 0.001, unpaired two-tailed t-test). However, the reactivation index only was significantly increased in AD patients in the CDR2 stage (Figure 2B(Fig. 2), p< 0.001, unpaired two-tailed t-test). Additionally, GPx activity was decreased in patients from all AD-CDR stages when compared to the control group (Figure 2D(Fig. 2), p< 0.001, unpaired two-tailed t-test).

### Correlation

**Figure 3:**
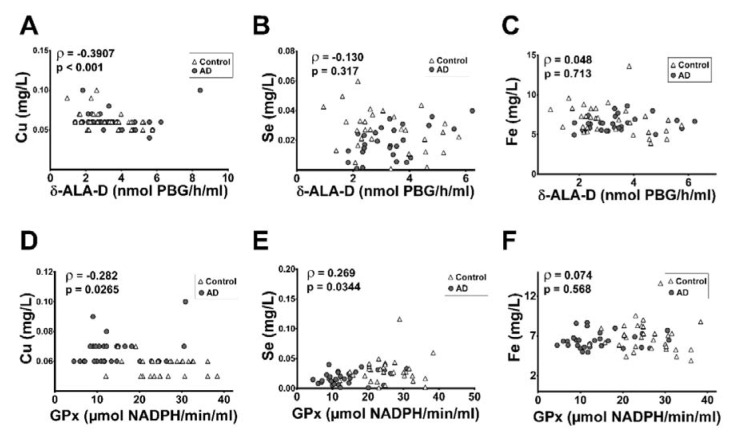
Correlations between the activity of δ-ALA-D (A, B and C) or GPx (D, E and F) with metals concentrations in the blood from control subjects and Alzheimer Disease (AD) patients according to Pearson correlation method. ρ: Pearson's correlation coefficient

We performed a correlation test using the δ-ALA-D activities and GPx data from all tested subjects (control group and AD patients) and blood measures of Fe, Cu and Se levels (previously published by Vaz et al., 2018[53]) using the Pearson's correlation analysis (Figure 3A-F(Fig. 3)). We detected a significant negative (weak) correlation between δ-ALA-D activity and blood concentrations of Cu (Figure 3A(Fig. 3), p<0.001, ρ= -0.3907, r^2^=0.19, Pearson correlation method). Furthermore, we found that GPx activity is negatively correlated with Cu levels (Figure 3D(Fig. 3), p<0.05, ρ= -0.282, r^2^=0.079) and positively correlated with Se levels in the blood (Figure 3E(Fig. 3), p<0.05, ρ= -0.269, r2=0.072). We also explored the correlation of both enzymes' activities with the metal levels in the blood in each AD-CDR stage separately (Table 1(Tab. 1)). Interestingly, it was detected a negative correlation between δ-ALA-D activity and Cu levels in patients on the CDR1 and CDR3 stages. A positive correlation was found between δ-ALA-D activity and Fe levels in AD-CDR2 patients and between δ-ALA-D activity and Se levels in AD-CDR3 patients. Moreover, we detected a positive correlation between GPx activity and Fe levels in patients in the AD-CDR1 stage.

## Discussion

In this study, we observed that AD patients showed an alteration in the δ-ALA-D and GPx activities. In addition, we observed a correlation between the inhibition of these enzymes and the blood levels of copper (Cu), iron (Fe), and selenium (Se).

The inhibition of the enzyme δ-ALA-D has been extensively studied because of its involvement in the oxidative stress biomarkers increase, being present in several pathological and physiological conditions, such as gestational diabetes mellitus (Rodrigues et al., 2018[45]), preeclampsia (de Lucca et al., 2016[14]), lung cancer (Zanini et al., 2014[57]), chronic renal failure (da Silva et al., 2007[13]), and type 2 diabetes (Bonfanti et al., 2011[7]).

Recently, Baierle et al. (2010[3], 2014[2]) suggested that the δ-ALA-D inhibition is an additional factor to the cognitive decline associated with aging. To the best of our knowledge, we demonstrated for the first time that AD patients showed significant δ-ALA-D inhibition when compared with age-matched control subjects. The inhibitory effects vary with disease progression: δ-ALA-D activity is maintained in AD-CDR1 patients while in AD-CDR2 and AD-CDR 3 patients, the enzyme was inhibited.

However, the enhanced reactivation index in the second stage of AD, but not in the third stage, may indicate that the enzyme δ-ALA-D oxidation state deteriorated with the AD progression. 

Metals can inhibit the enzyme δ-ALA-D and this effect has been described both *in vitro* and *in vivo *studies (Baierle et al., 2010[3]; Klimaczewski et al., 2018[26]; Mesquita et al., 2016[31]; Oliveira et al., 2014[33]; Pauza et al., 2005[37]; Peixoto et al., 2004[38]; Rocha et al., 2004[44], 2012[43]; Vargas et al., 2013[52]). In our study, we observed a negative correlation between the δ-ALA-D activity and the Cu levels in the blood. Interestingly, Baierle et al. (2010[3]) observed the same pattern in elderly women and Klimaczewski et al. (2018[26]), using *in silico* tools, observed that Cu(II) could enter in the δ-ALA-D active site, binding in the thiolate group of C135, the carboxyl moieties of D131 and E136, and the hydroxyl group of S179, interacting and oxidizing the thiol group. In this context, the reactivation of the enzyme by DTT, observed in our study, corroborates with these *in silico* findings. 

We also analyzed the enzyme GPx status in AD patients. GPx is an antioxidant selenoenzyme that catalyzes the reduction of hydrogen peroxide (H_2_O_2_) and lipid peroxides by glutathione (GSH) (Michiels et al., 1994[32]; Papp et al., 2007[36]). Alteration in the GPx activity has been reported in several human comorbidities, for instance, diabetes (Martín-Gallán et al., 2003[30]), renal disorders (El-far et al., 2005[19]), leukemia (Zuo et al., 2006[58]) and amyotrophic lateral sclerosis (Przedborski et al., 1996[40]). Regarding AD, there are inconsistent results concerning GPx activity in AD patients. Some authors observed that the enzyme is inhibited in the blood (serum and/or erythrocytes) of AD patients (Jeandel et al., 1989[25]; Padurariu et al., 2010[34]; Rinaldi et al., 2003[41]; Vural et al., 2010[54]) and other authors reported that GPx activity is maintained in AD patients (Bourdel-Marchasson et al., 2001[8]; Casado et al., 2007[9]; Ceballos-Picot et al., 1996[11]). In this study, we observed an inhibition in the GPx activity of AD patients. The conflicting results found in the literature may be explained by the Se status of the patients, since the GPx is a Se-dependent enzyme (Rotruck et al., 1973[46]). In fact, we observed a positive correlation between the enzyme activity and the Se levels. Taken together, ours results point out that GPx status evaluation may be better interpreted when combined with Se levels data.

## Conclusion

In summary, the decreased blood δ-ALA-D and GPx enzymes found in AD patients could be related to a deregulation in the blood homeostasis of Cu and Se, respectively. Furthermore, the pro-inflammatory and pro-oxidant scenario found in AD could be important factors contributing to deregulate δ-ALA-D and GPx activities as well. Our results indicate for the first time that blood δ-ALA-D activity is significantly inhibited in AD patients. Therefore, δ-ALA-D may be applied as an earlier AD marker.

## Notes

Cláudia Sirlene Oliveira and Juliana Sartori Bonini (Universidade Estadual do Centro-Oeste, Campus CEDETEG, Departamento de Farmácia, Guarapuava/PR, Brazil; Email: juliana.bonini@gmail.com) contributed equally as corresponding authors.

## Declaration of interest

The authors declare that they have no competing interests.

## Acknowledgements

The authors would like to thank the financial support from Coordination for Improvement of Higher Education Personnel (CAPES/PROEX- Finance Code 001), the National Council for Scientific and Technological Development (CNPq), the Rio Grande do Sul Foundation for Research Support (FAPERGS - Brazil), and the Instituto de Pesquisa Pelé Pequeno Príncipe.

## Supplementary Material

Supplementary data

## Figures and Tables

**Table 1:**
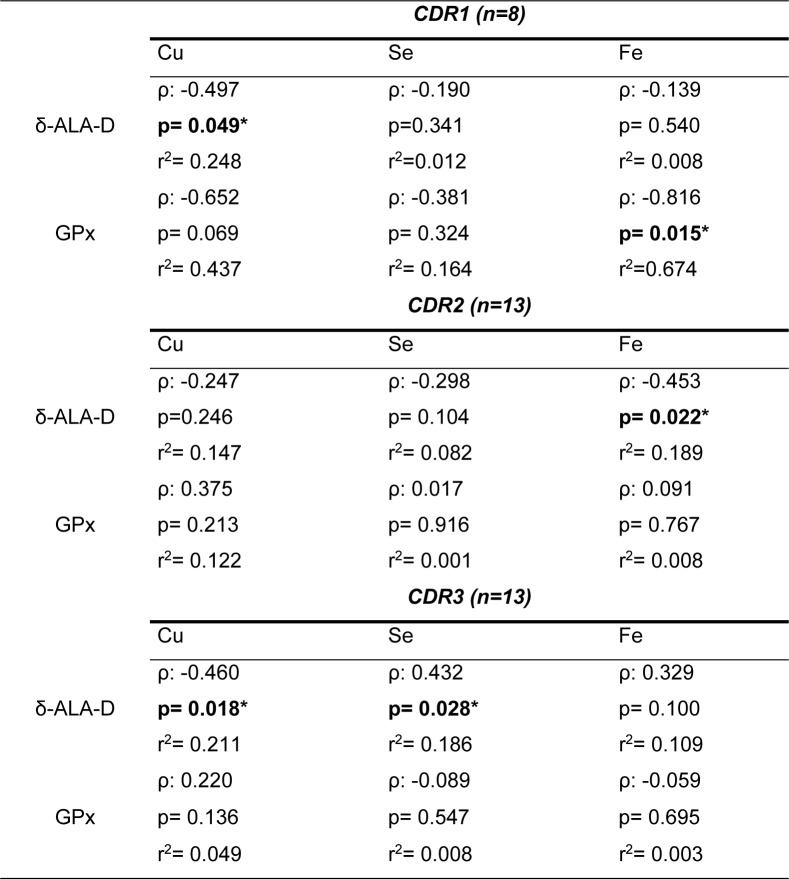
Correlations between the activity of δ-ALA-D or GPx with Cu, Se and Fe concentrations in the blood from Alzheimer Disease (AD) patients according to Pearson correlation method. ρ: Pearson's correlation coefficient; r^2^: coefficient of determination
