# Supplementary material for: Delta-Aminolevulinate dehydratase and glutathione peroxidase activity in Alzheimer's disease: a case-control study
Source: EXCLI J. 2019 Sep 25;18:866–75. doi: 10.17179/excli2019-1749 (PMC6806262; doi:10.17179/excli2019-1749)
Supplement: Supplementary data [file EXCLI-18-866-s-001.pdf]

EXCLI Journal 2019;18:866-875 – ISSN 1611-2156
Received: August 21, 2019, accepted: September 23, 2019, published: September 25, 2019

Supplementary data to:

Δ-AMINOLEVULINATE DEHYDRATASE AND GLUTATHIONE PEROXIDASE ACTIVITY IN ALZHEIMER'S DISEASE: A CASE-CONTROL STUDY

Quelen Iane Garlet[1], Maria Vaitsa Losh Haskel[2], Romaiana Picada Pereira[3], Weber Cláudio Francisco Nunes da Silva[4], João Batista Teixeira da Rocha[5], Cláudia Sirlene Oliveira[5,6,7*], Juliana Sartori Bonini[4*]

[1] Departamento de Farmacologia, Instituto de Ciências Biológicas, Universidade Federal de Rio Grande, Rio Grande/RS, Brazil
[2] Departamento de Fisiologia Humana, Universidade Federal do Rio Grande do Sul, Porto Alegre/RS90040-060, Brazil
[3] Departamento de Química, Universidade Estadual de Ponta Grossa, Ponta Grossa/PR, Brazil
[4] Universidade Estadual do Centro-Oeste, campus CEDETEG, Departamento de Farmácia, Guarapuava/PR, Brazil
[5] Departamento de Bioquímica e Biologia Molecular, Centro de Ciências Naturais e Exatas, Universidade Federal de Santa Maria, Santa Maria/RS, Brazil
[6] Programa Pós-Graduação Stricto Sensu em Biotecnologia Aplicada a Saúde da Criança e do Adolescente, Instituto de Pesquisa Pelé Pequeno Príncipe, Curitiba/PR, Brazil
[7] Faculdades Pequeno Príncipe, Curitiba/PR, Brazil

* Corresponding authors: Juliana Sartori Bonini (juliana.bonini@gmail.com), Cláudia Sirlene Oliveira (claudia.bioquimica@yahoo.com.br)

http://dx.doi.org/10.17179/excli2019-1749

This is an Open Access article distributed under the terms of the Creative Commons Attribution License (http://creativecommons.org/licenses/by/4.0/).

EXCLI Journal 2019;18:866-875 – ISSN 1611-2156
Received: August 21, 2019, accepted: September 23, 2019, published: September 25, 2019

Supplementary Table 1: δ-ALA-D and GPx activity raw data from control subjects and Alzheimer Disease (AD) patients are presented together with the blood concentrations of Cu, Se and Fe from the same samples previously published by Vaz et al., 2018

AD subject	CDR	MMSE	Age	Years of school	ALA-D (nm PBG/h/mL blood)	ALA-D + DTT (nm PBG/h/mL blood)	Reactivation index	GPx (µmol NADPH/min./mL blood)	Se* mg/L blood	Fe* mg/L blood	Cu* mg/L blood
P1	1	27	80	>4	3.90	5.38	15.06	9.97	0.0052	5.45	0.06
P2	1	16	84	≤4	3.23	7.90	34.30	8.04	-	-	0.05
P3	1	18	94	≤4	4.04	2.66	39.10	5.95	0.0083	6.33	0.06
P4	1	17	83	≤4	3.76	2.95	29.49	22.83	0.0309	6.90	0.05
P5	1	16	90	≤4	6.23	7.03	25.89	9.00	0.0398	8.59	0.06
P6	1	20	84	>4	8.44	6.43	42.30	14.63	0.0092	6.65	0.06
P7	1	26	79	>4	3.00	3.30	20.91	11.58	0.0284	8.68	0.10
P8	1	19	64	>4	2.79	1.67	0.00	26.37	0.0250	5.45	0.05
P9	2	9	70	≤4	3.32	3.33	34.83	9.00	0.0163	6.49	0.06
P10	2	14	82	≤4	4.69	4.02	16.92	24.60	0.0300	7.30	0.06
P11	2	14	70	≤4	4.80	3.28	17.99	18.17	0.0359	7.97	0.05
P12	2	10	63	≤4	4.76	4.21	42.28	10.45	0.0072	-	0.05
P13	2	14	75	≤4	5.76	3.13	26.52	14.63	-		0.06
P14	2	15	86	>4	2.41	2.74	65.33	14.95	0.0171	5.76	0.05
P15	2	9	60	≤4	3.42	3.30	20.91	6.91	0.0103	6.32	0.06
P16	2	9	77	≤4	3.30	3.26	30.98	30.55	0.0344	7.69	0.06
P17	2	14	85	≤4	1.83	4.86	45.27	7.07	0.0128	5.86	0.08
P18	2	11	83	≤4	2.34	3.04	42.43	9.65	0.0122	6.48	0.10
P19	2	11	76	≤4	5.58	1.60	12.50	16.56	0.0276	6.38	0.06
P20	2	9	58	≤4	2.13	4.60	25.00	8.36	0.0226	6.76	0.04
P21	2	15	80	>4	2.81	3.86	20.73	14.15	0.0161	-	0.07
P22	3	0	88	≤4	1.82	3.71	35.04	24.43	0.0051	5.55	0.06
P23	3	5	86	≤4	3.54	3.63	5.79	11.74	0.0056	4.95	0.06
P24	3	7	86	>4	3.78	4.42	25.34	12.22	0.0201	6.00	0.06
P25	3	0	79	≤4	2.39	2.01	8.96	30.87	0.0233	6.49	0.06
P26	3	7	65	≤4	2.86	3.46	32.37	9.32	0.0194	5.81	0.06
P27	3	7	67	≤4	3.54	6.50	14.15	13.34	0.0160	6.44	0.06
P28	3	2	67	>4	2.34	2.77	23.10	12.22	0.0016	5.62	0.06
P29	3	5	80	>4	2.17	5.90	52.37	14.63	0.0244	7.39	0.07
P30	3	0	81	>4	2.13	2.50	27.20	20.10	0.0011	5.43	0.06
P31	3	0	87	>4	3.30	4.62	23.38	10.93	0.0151	5.58	0.06
P32	3	7	80	>4	3.45	4.30	12.09	11.58	0.0267	8.26	0.06
P33	3	0	80	>4	2.39	3.36	28.87	4.50	0.0147	5.83	0.06
P34	3	3	77	>4	2.86	4.31	33.64	29.42	0.0345	-	0.06

ALA-D: δ-aminolevulinate dehydratase; CDR: Clinical Dementia Rating; Cu: Copper; DDT: dithiothreitol; Fe: Iron; GPx: glutathione peroxidase; MMSE: Mini-Mental State Examination; PBG: porphobilinogen; Se: Selenium. *Previously published by Vaz FNC, Fermino BL, Haskel MVL, Wouk J, de Freitas GBL, Fabbri R, et al. The relationship between copper, iron, and selenium levels and Alzheimer disease. Biol Trace Elem Res. 2018;181:185-91.

EXCLI Journal 2019;18:866-875 – ISSN 1611-2156
Received: August 21, 2019, accepted: September 23, 2019, published: September 25, 2019

Supplementary Table 1 (cont.): δ-ALA-D and GPx activity raw data from control subjects and Alzheimer Disease (AD) patients are presented together with the blood concentrations of Cu, Se and Fe from the same samples previously published by Vaz et al., 2018

Control subject	Paired CDR	MMSE	Age	Years of school	ALA-D (nm PBG/h/mL blood)	ALA-D + DTT (nm PBG/h/mL blood)	Reactivation index	GPx (µmol NADPH/min./mL blood)	Se* mg/L blood	Fe* mg/L blood	Cu* mg/L blood
C1	Controls paired with CDR1 patients	25	87.00	≤4	4.62	6.89	33.02	36.13	0.0019	3.90	0.06
C2		29	83.00	>4	5.26	4.55	0.00	20.77	0.0312	6.82	0.06
C3		21	83.00	≤4	4.42	2.54	0.00	27.54	0.0436	7.30	0.06
C4		29	63.00	>4	2.30	4.79	52.04	19.13	0.0208	5.87	0.09
C5		22	79.00	≤4	3.98	5.75	30.75	22.58	0.0108	4.94	0.07
C6		22	86.00	≤4	2.42	4.22	42.67	20.32	0.0326	7.19	0.07
C7		24	93.00	≤4	3.78	3.86	1.97	20.77	0.0476	-	0.06
C8		21	89.00	≤4	4.54	6.47	29.82	32.52	0.0207	5.26	0.06
C9	Controls paired with CDR2 patients	30	77.00	>4	4.57	3.08	0.00	32.70	0.0122	4.41	0.05
C10		22	78.00	≤4	5.19	4.17	14.03	26.77	0.0254	5.46	0.06
C11		22	85.00	≤4	1.62	5.02	0.00	23.03	0.0495	9.57	0.07
C12		28	66.00	>4	2.08	7.28	38.54	24.87	0.0051	7.28	0.05
C13		28	69.00	>4	5.21	5.22	8.09	20.77	0.0117	4.41	0.05
C14		28	69.00	>4	3.71	3.84	16.38	14.90	0.0275	7.96	0.06
C15		22	77.00	≤4	2.61	5.14	15.19	30.70	0.0273	7.06	0.06
C16		26	82.00	≤4	1.68	3.45	0.00	32.06	0.0289	-	0.07
C17		22	81.00	≤4	3.86	5.15	16.89	27.22	0.0269	6.51	0.06
C18		23	79.00	≤4	3.83	4.76	18.91	28.90	0.1160	13.60	0.05
C19		23	57.00	≤4	2.14	4.55	15.95	30.03	0.0267	5.98	0.05
C20		25	85.00	≤4	5.75	2.61	18.26	15.80	0.0222	6.18	0.05
C21		22	79.00	≤4	2.17	3.98	0.00	36.13	0.0166	5.28	0.05

ALA-D: δ-aminolevulinate dehydratase; CDR: Clinical Dementia Rating; Cu: Copper; DDT: dithiothreitol; Fe: Iron; GPx: glutathione peroxidase; MMSE: Mini-Mental State Examination; PBG: porphobilinogen; Se: selenium. *Previously published by Vaz FNC, Fermino BL, Haskel MVL, Wouk J, de Freitas GBL, Fabbri R, et al. The relationship between copper, iron, and selenium levels and Alzheimer disease. Biol Trace Elem Res. 2018;181:185-91.

EXCLI Journal 2019;18:866-875 – ISSN 1611-2156
Received: August 21, 2019, accepted: September 23, 2019, published: September 25, 2019

Supplementary Table 1 (cont.): δ-ALA-D and GPx activity raw data from control subjects and Alzheimer Disease (AD) patients are presented together with the blood concentrations of Cu, Se and Fe from the same samples previously published by Vaz et al., 2018

Control subject	Paired CDR	MMSE	Age	Years of school	ALA-D	ALA-D + DTT	Reactivation index	GPx	Se*	Fe*	Cu*
C22	Controls paired with CDR3 patients	26	76.00	≤4	2.17	5.65	30.90	38.38	0.0597	8.78	0.06
C23		25	79.00	≤4	3.34	6.17	47.63	23.04	0.0009	5.67	0.06
C24		29	84.00	>4	2.69	4.13	2.10	20.32	0.0372	7.11	0.07
C25		28	65.00	>4	2.43	6.89	45.44	24.42	0.0271	8.26	0.07
C26		24	63.00	≤4	2.30	5.65	0.00	30.25	0.0213	5.34	0.06
C27		28	80.00	>4	0.95	8.75	3.49	24.83	0.0425	8.16	0.09
C28		25	83.00	≤4	2.61	6.09	50.71	20.32	0.0411	8.29	0.10
C29		23	79.00	≤4	2.25	3.58	22.12	31.60	0.0330	7.31	0.06
C30		22	79.00	≤4	2.66	4.87	31.85	30.24	0.0211	5.69	0.06
C31		22	83.00	≤4	1.75	4.95	5.25	23.48	0.0325	8.30	0.06
C32		23	59.00	≤4	1.40	4.48	0.00	12.06	0.0131	5.96	0.06
C33		23	79.00	≤4	3.45	6.09	21.89	24.38	0.0358	6.85	0.06
C34		24	75.00	≤4	3.06	5.95	3.28	24.83	0.0399	9.02	0.06

ALA-D: δ-aminolevulinate dehydratase; CDR: Clinical Dementia Rating; Cu: Copper; DDT: dithiothreitol; Fe: Iron; GPx: glutathione peroxidase; MMSE: Mini-Mental State Examination; PBG: porphobilinogen; Se: selenium. *Previously published by Vaz FNC, Fermino BL, Haskel MVL, Wouk J, de Freitas GBL, Fabbri R, et al. The relationship between copper, iron, and selenium levels and Alzheimer disease. Biol Trace Elem Res. 2018;181:185-91.
